# Structure of Some Green Tea Catechins and the Availability of Intracellular Copper Influence Their Ability to Cause Selective Oxidative DNA Damage in Malignant Cells

**DOI:** 10.3390/biomedicines10030664

**Published:** 2022-03-12

**Authors:** Mohd Farhan, Asim Rizvi, Aamir Ahmad, Mohammad Aatif, Mir Waqas Alam, Sheikh Mumtaz Hadi

**Affiliations:** 1 Department of Basic Sciences, Preparatory Year Deanship, King Faisal University, Al-Ahsa 31982, Saudi Arabia; 2 Department of Kulliyat, Faculty of Unani Medicine, Aligarh Muslim University, Aligarh 202002, India; rizvirizviasim@gmail.com; 3 Interim Translational Research Institute, Academic Health System, Hamad Medical Corporation, Doha 3050, Qatar; aahmad9@hamad.qa; 4 Department of Public Health, College of Applied Medical Sciences, King Faisal University, Al-Ahsa 31982, Saudi Arabia; maahmad@kfu.edu.sa; 5 Department of Physics, College of Science, King Faisal University, Al-Ahsa 31982, Saudi Arabia; wmir@kfu.edu.sa; 6 Department of Biochemistry, Faculty of Life Sciences, Aligarh Muslim University, Aligarh 202002, India; smhadi1946@gmail.com

**Keywords:** cancer, DNA damage, copper, catechins, apoptosis

## Abstract

The possible roles of elevated endogenous copper levels in malignant cells are becoming increasingly understood at a greater depth. Our laboratory has previously demonstrated that tea catechins have the ability to mobilize endogenous copper and undergo a Fenton-like reaction that can selectively damage cancer cells. In this communication, by using a diverse panel of malignant cell lines, we demonstrate that the ability of the catechin family [(−)-epigallocatechin-3-gallate (EGCG), (−)-epigallocatechin (EGC), (−)-epicatechin (EC), and (+)-catechin (C)] to induce apoptosis is dependent on their structure. We further confirm that reactive oxygen species (ROS) are the terminal effectors causing copper-mediated DNA damage. Our studies demonstrate the role of cellular copper transporters CTR1 and ATP7A in the survival dynamics of malignant cells post-EGCG exposure. The results, when considered together with our previous studies, highlight the critical role that copper dynamics and mobilization plays in cancer cells and paves the way for a better understanding of catechins as nutraceutical supplements for malignancies.

## 1. Introduction

The progression of clinical malignancies is a complex phenomenon, which is regulated by a large number of factors, that can influence the promotion and progression of disease. Dietary constituents, particularly polyphenolic compounds [1], have been shown to affect malignancies by causing selective cell death of malignant cells [2,3]. Catechins are a class of polyphenols derived primarily from tea [4], which include (−)-epigallocatechin-3-gallate (EGCG), (−)-epigallocatechin (EGC), (−)-epicatechin gallate (ECG), (+)-gallocatechin (GC), (−)-epicatechin (EC), and (+)-catechin (C). Experimental evidence from our lab [5,6] and those of others [7,8] has shown that EGCG is the most potent amongst this class of molecules in causing malignant cell death. There is enough literature to suggest that, in cell lines, catechins can affect a variety of metabolic and signaling pathways [9]. These molecular events may result in cancer cell growth inhibition, apoptosis, inhibition of invasion, angiogenesis, and metastasis [9,10]. The inhibition of tumorigenesis by catechins has also been demonstrated in different animal models, including those for cancer of the skin, lung, esophagus, stomach, colon, bladder, liver, pancreas, prostate, and mammaries [9]. It is worth mentioning that plant-derived polyphenols, including catechins, are capable of causing selective cell death of malignant cells, while sparing normal cells [11]. Thus, catechins may be considered as potential anticancer compounds.

Several malignant cell types are sensitive to plant-derived polyphenolic compounds; therefore, it is reasonable to presume that the molecular target(s) for the selective cell death of malignant cells is (are) a metabolic feature common to all malignancies [12]. In this regard, elevated copper levels have been shown to be a feature common to most malignancies [13]. Heterochromatic copper chelators have been developed and have shown promise in this area [14,15,16]. This phenomenon has been extensively reviewed [17,18,19], and malignancies, irrespective of their tissue of origin, have been shown to harbor elevated levels of copper compared to normal tissue. Furthermore, this selective elevation of copper levels is demonstrated in both solid tumors as well as in blood malignancies [20].

We have previously shown that the prooxidant activity of plant-derived polyphenols, by which they mediate their selective anticancer action, is a consequence of the selective elevation of copper levels in malignant cells as compared to non-malignant controls [3,21]. Our studies demonstrate that plant-derived polyphenolics react with cellular copper in the vicinity of DNA by a Fenton-like reaction, resulting in ROS production. These ROSs in turn damage the genomic DNA of malignant cells, resulting in apoptosis-like cell death.

**Figure 1:**
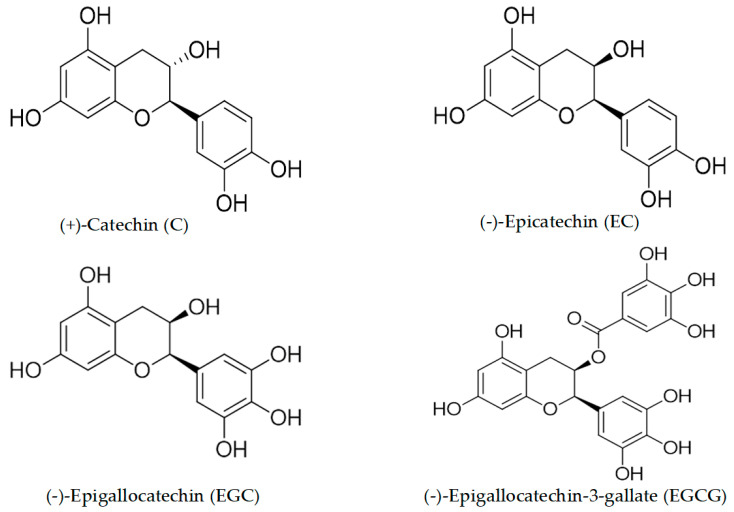
Structures of some tea catechins.

In the present work, we establish the potent oxidative, damage-inducing ability of some catechins from green tea. We show that such abilities of tea catechins in malignant cells are dependent upon the cellular bioavailability of copper and its redox recycling. The structures of green tea catechins used in this study are shown in Figure 1.

## 2. Materials and Methods

Cell lines and Reagents: Immortalized non-transformed breast cell line MCF-10A and cancer lines, PC3, MDA-MB-231, BxPC-3, and MiaPaCa-2, were obtained from ATCC (Manassas, VA, USA). MDA-MB-231, BxPC-3, and MiaPaCa-2 cell lines were maintained in DMEM (Invitrogen, Carlsbad, CA, USA), while PC3 cells were maintained in RPMI (Invitrogen, Carlsbad, CA, USA). Both of these media were supplemented with 10% fetal bovine serum (FBS), 100 units/mL penicillin, and 100 µg/mL streptomycin. All cells were cultured in a 5% CO_2_-humidified atmosphere at 37 °C. Stock solutions of C, EC, EGC, and EGCG (50 mM) were made in DMSO, and small aliquots were stored at −20 °C. The stock solutions of different chelators of the metal ions—neocuproine (Neo)/desferoxamine mesylate (DM)/histidine (His)—were made in PBS at a final concentration of 50 mM and were always made fresh prior to experiments. A normal breast epithelial cell line, MCF-10A, was propagated in DMEM/F12 (Invitrogen, Carlsbad, CA, USA) supplemented with 5% horse serum, 20 ng/mL EGF, 0.5 µg/mL hydrocortisone, 0.1 µg/mL cholera poison, 10 µg/mL insulin, 100 units/mL penicillin, and 100 µg/mL streptomycin in a 5% CO_2_ climate at 37 °C.

MCF-10A + Cu cells are MCF-10A cells that were cultured in their normal culture media (above) with extra supplementation of 25 µM CuCl_2_ for a month.

Cell growth inhibition studies by 3-(4,5-Dimethylthiazol-2-yl)-2,5 diphenyltetra-zolium bromide (MTT) assay: Cells were seeded at a density of 2 × 10^3^ cells per well in 96-well microtiter culture plates. After overnight incubation, the normal growth medium was removed and replaced with a fresh medium containing different concentrations of respective catechins diluted from a 50 mM stock. Various chelators were added in individual assays, as mentioned in respective experiments. After 3 days of incubation, 25 µL of 3-(4,5-Dimethylthiazol-2-yl)-2,5-diphenyltetrazolium bromide (MTT) solution (5 mg/mL in PBS) was added to each well and incubated further for 2 h at 37 °C. After completion of the 2 h incubation, the supernatant was removed and MTT formazan, framed by metabolically viable cells, was broken down in DMSO (100 µL) by blending for 30 min on a gyratory shaker. The absorbance was estimated at 595 nm on an Ultra Multifunctional Microplate Reader (TECAN, Durham, NC, USA). Every treatment had eight recreate wells and the measure of DMSO in the response blend never surpassed 0.1%. Additionally, each examination was repeated at least three times.

Histone/DNA ELISA for detection of apoptosis: The Cell Death Detection ELISA Kit (Roche, Palo Alto, CA, USA) was utilized to identify apoptosis in growth cells treated with various catechins.

Cells were treated with polyphenolic mixes, or DMSO control, for 72 h. After treatment, the cytoplasmic histone and DNA from cells were isolated and incubated in the microtiter plate modules covered with anti-histone antibody. The peroxidase-conjugated anti-DNA antibody was utilized for the of immobilized histone/DNA followed by color advancement with ABTS substrate for peroxidase. The spectrophotometric absorbance of the examples was read by using Ultra Multifunctional Microplate Peruser (TECAN, Durham, NC, USA) at 405 nm.

The reactions were additionally performed with particular metal ion chelators. DM (50 µM) was utilized for the chelation of Fe (II) particles, His (50 µM) was utilized for Zn (II), and Neo (50 µM each) was utilized for the chelation of Cu (II) particles. Free radical scavengers (catalase 20 µg/mL, superoxide dismutase (SOD) 20 µg/mL, and thiourea (TU) 0.1 mM) were utilized to examine the role of reactive oxygen species in the intracellular reaction of copper with various tea catechins.

Cell migration assay: A cell migration assay was performed by utilizing 24-well transwell permeable supports with 8 mm pores (Corning, NY, USA). Cells were suspended in a serum-free medium and seeded into the transwell embeds. The bottom wells were loaded with media containing complete media.

After 24 h, cells were stained with 4 mg/mL calcein AM (Invitrogen, Carlsbad, CA, USA) in PBS at 37 °C for 1 h and detached from inserts by trypsinization.

The fluorescence of the migrated cells was read in ULTRA Multifunctional Microplate Peruser (TECAN, Durham, NC, USA). The cells were grown in the presence and absence of EGCG (50 µM) with or without neocuprione (50 µM).

Real-time reverse transcriptase PCR: Total RNA was isolated by using the TRIzol reagent (Invitrogen, Carlsbad, CA, USA) according to the manufacturer’s instructions. Real-time PCR was used to quantify mRNA expressions. Sequences of primers for Ctr1 (forward: 5′-GCT GGA AGA AGG CAG TGG TA-3′; reverse: 5′-AAA GAG GAG CAA GAA GGG ATG-3′), ATP7A (forward: 5′-ACG AAT GAG CCG TTG GTA GTA-3′; reverse: 5′-CCT CCT TGT CTT GAA CTG GTG-3′) and GAPDH (glyceraldehyde-3-phosphate dehydrogenase) (forward: 5′-TGG GTG TGA ACC ATG AGA AGT-3′; reverse: 5′-TGA GTC CTT CCA CGA TAC CAA-3′) were the same as reported earlier [22,23], and the amount of RNA was normalized for GAPDH expression.

siRNA (small interfering RNA) transfection: siRNA transfections were performed, as described previously [23]. siRNA specific to ctr1 was purchased from Santa Cruz Biotechnology, Inc (Santa Cruz, Dallas, TX, USA). Scrambled siRNA was used as a nonspecific control. Transfections were performed by using Lipofectamine RNA iMAX Transfection Reagent (Invitrogen, Carlsbad, CA, USA) following the manufacturer’s instruction. Ctr1 was silenced by siRNA for 48 h prior to the assay.

## 3. Results

### 3.1. Catechins Inhibit Growth and Induce Apoptosis in Different Types of Cancer Cells

**Figure 2:**
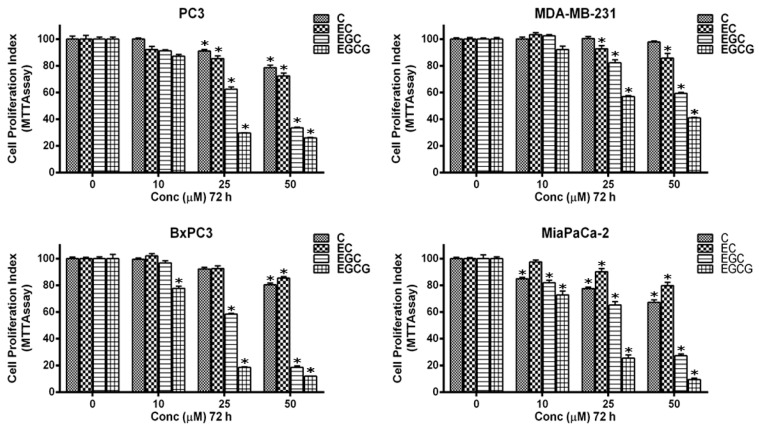
Effect of C, EC, EGC, and EGCG on cell proliferation in various cancer cell lines as detected by MTT assay. Cells from PC3, MDA-MB-231, BxPC3, and MiaPaCa-2 cancer cell lines were incubated with indicated concentrations of C, EC, EGC, and EGCG for 72 h. The effect on cell proliferation was detected by performing an MTT assay, as described in the Materials and Methods section. All results are expressed as percentage of control ± S.E. of triplicate determinations. * *p* < 0.01 when compared to respective untreated control.

In order to examine cancer cell growth inhibition by catechins, different cancer cell lines, namely PC3 (prostate), MDA-MB-231 (breast), and BxPC3 and MiaPaCa-2 (pancreas), were subjected to treatment with varying concentrations of C, EC, EGC, and EGCG by MTT assay (Figure 2). All catechins caused a clear concentration-dependent inhibition. However, the inhibition was found to be much greater in the case of EGCG than compared to EGC, EC, and C.

**Figure 3:**
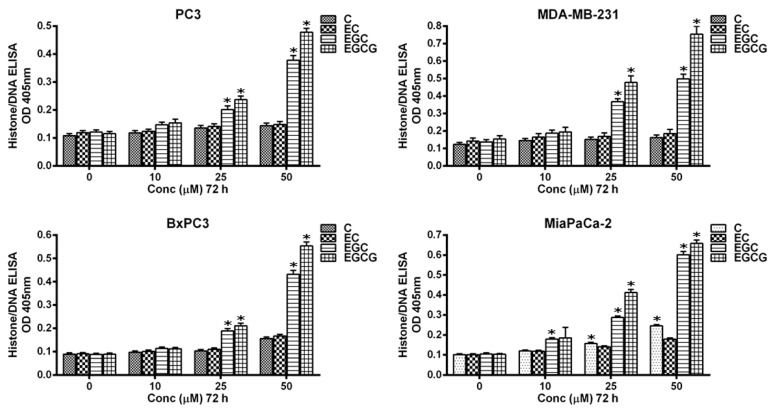
Apoptosis induction by C, EC, EGC, and EGCG in different cancer cell lines. The Cell Death Detection ELISA Kit (Roche, Palo Alto, CA, USA) was used to detect apoptosis in cells from different cancer cell lines after incubation for 72 h with increasing concentrations of C, EC, EGC, and EGCG as indicated in the figure and described in the Materials and Methods section. Values reported are ±S.E. of three independent experiments. * *p* value < 0.01 when compared to control.

To further confirm these results, the induction of apoptosis by C, EC, EGC, and EGCG was assayed by Histone/DNA ELISA (Figure 3). EGCG was found to be the most potent compound, which was in agreement with our previous results. EGCG was also the most effective inducer of apoptosis, followed by EGC, EC, and C. Taken together, these results demonstrated a dose-dependent cytotoxic action of catechins. Additionally, since EGCG was the most effective compound, we only utilized this compound for further mechanistic investigations.

### 3.2. Copper Chelation Inhibits EGCG-Induced Growth Inhibition and Apoptosis

**Figure 4:**
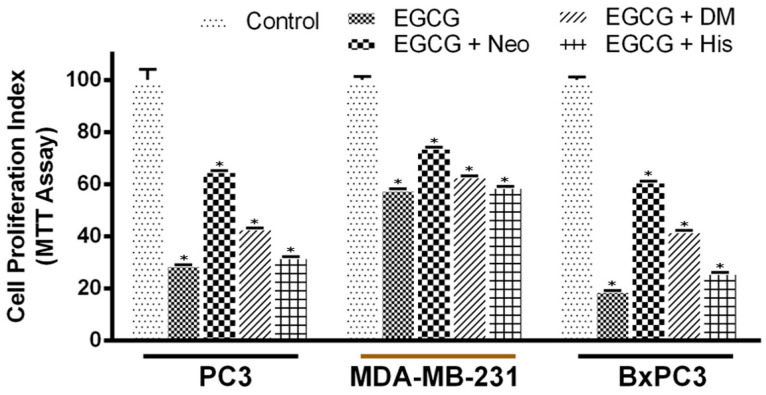
Effects of various metal-specific chelators on the antiproliferative activity of EGCG in three different cancer cell lines. PC3, MDA-MB-231, and BxPC3 cancer cells were treated with 25 µM of EGCG either alone or in the presence of copper chelator neocuproine (Neo), iron chelator desferrioxamine mesylate (DM), or zinc chelator histidine (His), as indicated in the figure. The concentration of metal chelators used was 50 µM. An MTT assay was performed after 72 h of treatment as described in the Materials and Methods section. Values reported are ±S.E. of three independent experiments. * *p* value < 0.01 when compared to control.

We have previously demonstrated that the membrane-permeable copper chelator neocuproine is able to inhibit catechin-induced oxidative breakages of cellular DNA in lymphocytes [5], suggesting the association of endogenous copper in the process. We questioned whether this phenomenon was relevant to cancer cells as well. Therefore, we replicated the study in malignant cells and observed that only the copper chelator, Neo, was able to protect PC3, MDA-MB-231, and BxPC-3 cells to a significant extent, against the growth-inhibitory action of EGCG (Figure 4). On the other hand, DM and H (iron and zinc chelators, respectively) failed to demonstrate such effects to any significant degree, except in the case of PC3 and BxPC3 cells where DM and His also showed some protective effect on EGCG-induced growth inhibition. However, this was still less than the inhibition with Neo. 

**Figure 5:**
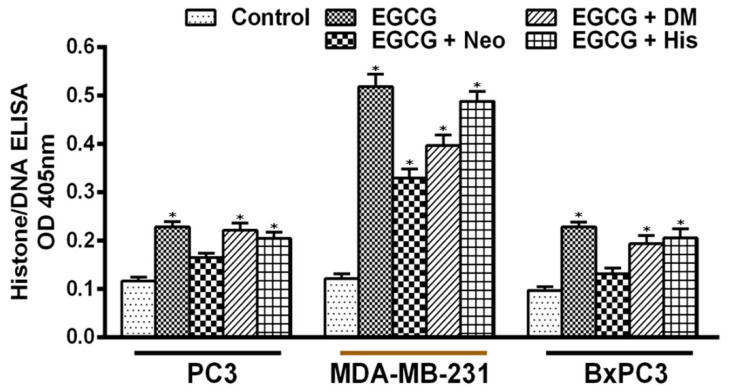
Effects of different metal chelators on apoptosis induction by EGCG in three different cancer cell lines. PC3, MDA-MB-231, and BxPC3 cancer cells were treated with 25 µM of EGCG either alone or in the presence of copper chelator neocuproine (Neo), iron chelator desferrioxamine mesylate (DM), or zinc chelator histidine (His), as indicated in the figure. The concentration of metal chelators used was 50 µM. An MTT assay was performed after 72 h of treatment as described in the Materials and Methods section. Values reported are ±S.E. of three independent experiments. * *p* value < 0.01 when compared to control.

The effect of different metal chelators was additionally tested against EGCG-induced apoptosis (Figure 5). Copper chelator Neo provided a significant degree of protection. This protection was not observed when either an iron or zinc chelator was utilized, thus confirming the conclusion that the anticancer mechanism of EGCG involves the mobilization of endogenous copper.

### 3.3. Apoptosis of Cancer Cells Induced by EGCG Is Mediated by ROS

DNA breakages by prooxidant anticancer compounds [24,25] involve the generation of ROS [5]. With the specific end goal of verifying whether the catechin-induced DNA damage in cancer cell lines also involved ROS, the effect of various scavengers of ROS (for example, catalase, thiourea, and superoxide dismutase) on EGCG-induced apoptosis of cancer cells was examined. All three ROS scavengers caused moderate to considerable suppression of EGCG-induced apoptotic activity in various cancer cell lines tested (Table 1), with TU showing the highest level of suppression. These results reaffirmed the role of ROS as effectors of catechin-induced apoptosis [5], possibly by a Fenton- type, biologically active reaction, as previously described [13,26,27,28].

### 3.4. Copper Chelation Abrogates EGCG-Induced Inhibition of Migration by Malignant Cells

**Figure 6:**
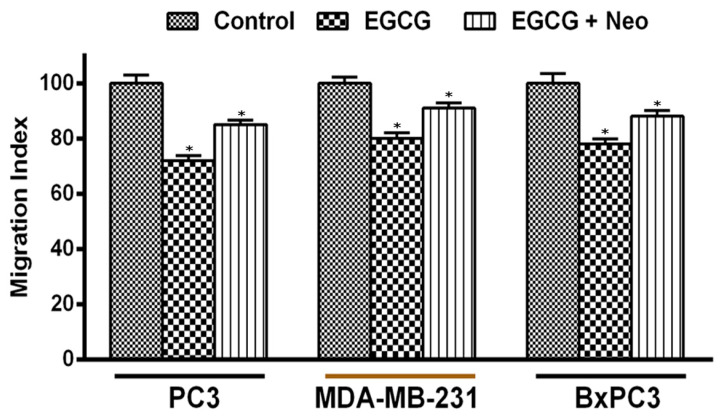
Effect of EGCG on migration of PC3 (prostate), MDA-MB-231 (breast), and BxPC3 (pancreatic) cancer cells in the presence of copper chelating agent neocuproine. A cell migration assay was performed by using 24-well transwell permeable supports with 8 mm pores (Corning, NY, USA) as described in the Materials and Methods section. The cells were grown in the presence and absence of EGCG (50 µM) with or without neocuprione (50 µM). The fluorescence of the migrated cells was read in an Ultra Multifunctional Microplate Reader (TECAN, Durham, NC, USA). Values reported are ±S.E. of three independent experiments. * *p* value < 0.01 when compared to control.

Migration and metastatic invasions to secondary sites are a characteristic feature of malignant cells. We observed that EGCG inhibited the migratory potential of PC3, MDA-MB-231, and BxPC3 cells (Figure 6), thereby making the cells less prone to metastasis. Interestingly, when copper was chelated from the cells by the membrane-permeable copper chelator neocuprione in the presence of EGCG, the cells regained their metastatic potential, thereby implicating the role of cellular copper in the EGCG-induced inhibition of migration of malignant cells.

### 3.5. Supplementation with Copper Sensitizes Normal Breast Epithelial Cells to Antiproliferative Action of EGCG

**Figure 7:**
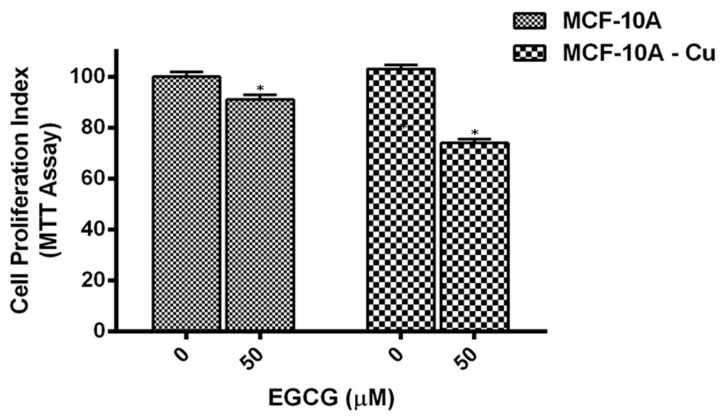
Effect of EGCG on inhibition of cell proliferation in MCF-10A (normal breast epithelial cells) and MCF-10A cells cultured in media supplemented with Cu (II) (MCF-10A-Cu). Both MCF-10A and MCF-10A-Cu (normal cells cultured in a medium containing 25 µM CuCl_2_) were subjected to treatment with EGCG for 72 h at concentrations, as indicated in the figure. Cell proliferation was subsequently estimated by MTT assay as described in the Materials and Methods section. Values reported are ±S.E. of three independent experiments. * *p* value < 0.01 when compared to control.

Normal (non-malignant) breast epithelial cells, MCF-10A, were cultured in media supplemented with 25 µM copper. At the point when such copper-supplemented cells (MCF-10A + Cu) were treated with EGCG, a decrease in cell proliferation was observed, which was significant in contrast to non-copper-supplemented MCF-10A cells (Figure 7).

Since malignant transformation is accompanied by a drastic rise in intracellular levels of malignant cells [17,19], it is reasonable to infer that the EGCG-induced inhibition of growth of malignant cells is a consequence of its interaction with cellular copper. The supplementation of non-malignant epithelial cells with exogenous copper results in the sensitization of these non-malignant cells to catechin-induced cell growth inhibition.

### 3.6. EGCG Inhibits the Expression of Copper Transporters Ctr1 and ATP7A

**Figure 8:**
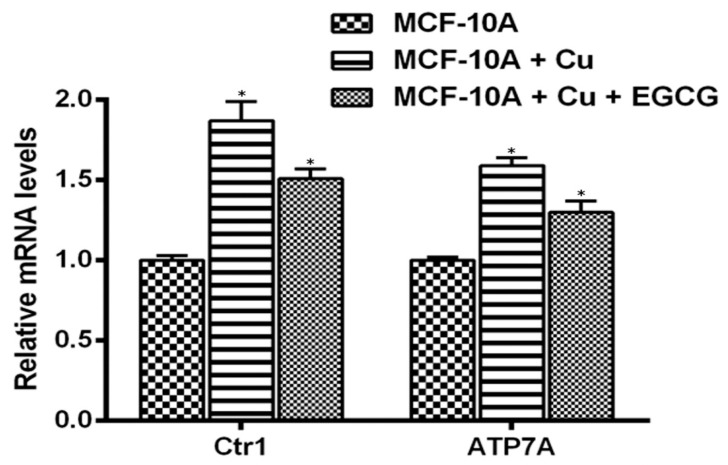
Elevated mRNA transcript levels of copper transporters Ctr1 and ATP7A in MCF-10A-Cu cells, relative to the parental MCF-10A cells, and the effect of EGCG. Total RNA was isolated by using TRIzol reagent (Invitrogen, Carlsbad, CA, USA) according to the manufacturer’s instructions. A real-time PCR was used to quantify Ctr1 and ATP7A mRNA expression as described in the Materials and Methods section. Only MCF-10A-Cu (normal MCF-10A cells cultured in a medium containing 25 µM CuCl_2_), with elevated mRNA expression of copper transporters, was subjected to treatment with EGCG (50 µM) to assess the effect of EGCG on mRNA expression. Values reported are ±S.E. of three independent experiments. * *p* value < 0.01 when compared to control.

We observed that EGCG-induced growth inhibition is a consequence of its interaction with intracellular copper both in malignant cells (Figure 4 and Figure 5) and in non-malignant epithelial cells when grown in a copper-supplemented medium (Figure 7). Since malignant cells have a higher expression of copper transporter Ctr1 [19], we next checked if copper supplementation resulted in increased copper transporter expression in non-malignant epithelial cells. We found that copper supplementation in the growth medium of MFC-10A cells resulted in a marked increase in the expression of the copper transporters Ctr1 and ATP7A [29] (Figure 8). Further supplementation of EGCG to the medium resulted in a decrease in the expression of both the copper transporters, demonstrating an effect of EGCG on copper metabolism in cancer cells.

### 3.7. Targeted Silencing of CTR1 in MCF-10A Cells Grown in Copper Supplemented Medium Reduces EGCG-Induced Inhibition of Proliferation

**Figure 9:**
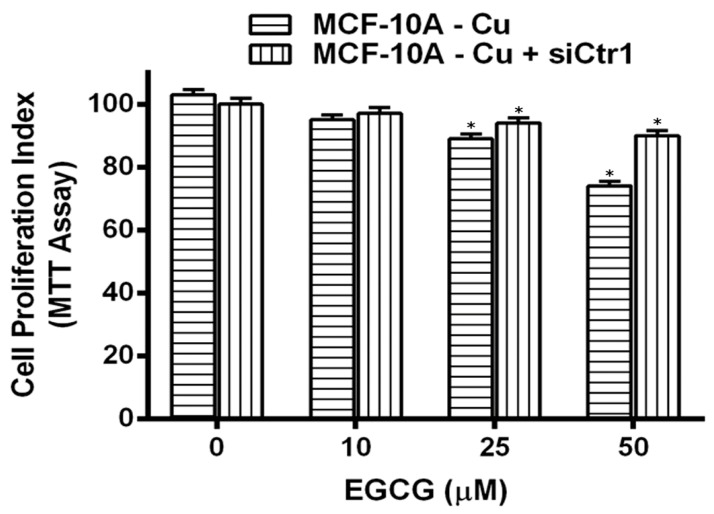
Elevated mRNA transcript levels of copper transporters Ctr1 and ATP7A in MCF-10A-Cu cells, relative to the parental MCF-10A cells, and the effect of EGCG. Total RNA was isolated by using TRIzol reagent (Invitrogen, Carlsbad, CA, USA) according to the manufacturer’s instructions. Real-time PCR was used to quantify Ctr1 and ATP7A mRNA expression as described in the Materials and Methods section. Only MCF-10A-Cu (normal MCF-10A cells cultured in a medium containing 25 µM CuCl_2_), with elevated mRNA expression of copper transporters, was subjected to treatment with EGCG (50 µM) to assess the effect of EGCG on mRNA expression. Values reported are ±S.E. of three independent experiments. * *p* value < 0.01 when compared to respective control.

To confirm the important role of copper in EGCG-induced growth inhibition, we silenced copper transporter ctr1 (Figure 9) by using specific siRNA. Ctr1 mediates copper uptake in cells and, as shown above (Figure 8), its expression increases the susceptibility of MCF-10A cells relative to EGCG-induced growth inhibition. We found that the silencing of copper transporter Ctr1 resulted in reduced sensitivity to EGCG of MCF-10A cells grown in a copper-enriched medium. This finding clearly indicates that EGCG interacts with cellular copper and that cellular copper is crucial for the growth-inhibitory action of EGCG against cancer cells.

## 4. Discussion

Our laboratory has demonstrated extensively that the prooxidant action of plant-derived polyphenols, as a consequence of their interaction with intracellular copper [2,3,21,30], in addition to the resultant redox signaling [31], is one of the mechanisms by which polyphenols exert their selective cytotoxic action. We have also previously proposed that the number and positions of the hydroxyl groups in the catechin skeleton are important for determining the degree of copper-mediated cellular DNA breakage [5] and that ortho-hydroxyls play an important role, possibly by providing a chelation mechanism of Cu (II) and its reduction to Cu (I). The present study confirms this by an experimental demonstration of the activity of tea catechins (Figure 1) against cancer cells, which conforms to the following order: EGCG > EGC > EC > C. A similar mechanism for the prooxidant action of Vitamin D in the presence of copper in malignant cells has also been demonstrated [13,26,27,28].

The observation that “normal” breast epithelial MCF-10A cells are refractory to the cytotoxic effect of catechins, as compared to the tumorigenic breast MDA-MB-231 cells, is an interesting observation, clearly demonstrating the cancer cell selectivity of tea catechins in exerting their cytotoxic effect. Our novel observation that MCF-10A cells acquire sensitivity to tea catechins-induced cytotoxicity, when cultured in the presence of copper, underpins the crucial role that cellular copper plays in catechins-mediated physiological reactions resulting in cell death.

The physiological role of copper in malignancies is still not very well known. However, there is evidence to support the role of increased levels of copper in tumor angiogenesis [32] and protein aggregation [20]. Our hypothesis [3] that plant-derived polyphenols, specifically tea catechins, interact with intracellular copper and mediate oxidative DNA breakage has been experimentally validated with considerable success [5,6,33]. In this regard, the present study further strengthens our hypothesis. The ability of EGCG to inhibit breast tumor angiogenesis has been demonstrated [34,35], and it is worth speculating that such anti-angiogenesis actions of EGCG involve copper mobilization and the resulting prooxidant effect, which is an idea that needs to be further tested.

Both of the copper transporters that we tested in the present study, CTR1 and ATP7A, were found to be upregulated when normal epithelial cells were cultured in the presence of copper. Furthermore, EGCG could inhibit the expression of these transporters. Thus, the acquired sensitivity of epithelial cells to EGCG action correlated with the expression of copper transporters. This fact adds another level of regulation to our hypothesis, whereby EGCG not only interacts with copper and results in oxidative DNA damage but also inhibits copper transporters, thereby crippling the copper metabolism of the ”transformed” cell(s) that seems to be essential for the survival of these cells [17,36]. We were further able to confirm our results through an experimental setup involving the inhibition of the expression of representative copper transporter ctr1 by siRNA. Such silencing of ctr1 abrogated the EGCG sensitivity of MCF-10A grown with copper supplementation, clearly demonstrating and validating that copper is essential for the selective cell death induced by EGCG. In a nutshell, it may be concluded that the structure of tea catechins and the availability of intracellular copper influence their ability to cause oxidative DNA damage in malignant cells. We have presented novel results to establish the crucial role of intracellular copper levels, made possible by copper transporters, in the anticancer action of tea catechins in particular and the plant-derived polyphenols in general. This provides a new dimension for the design of future mechanism-based studies to target the tumor microenvironment for the desired efficacy of non-toxic anticancer compounds, such as tea-derived catechins.

## Figures and Tables

**Table 1:** Effect of ROS scavengers on EGCG-induced apoptotic activity in three different cancer cell lines. Along with EGCG, cancer cells were incubated with various ROS scavengers, namely TU, 700 µM Thiourea; Cat, 100 mg/mL catalase; and SOD, 100 mg/mL superoxide dismutase. The effect on apoptosis was assessed by using Histone/DNA ELISA as described in the Materials and Methods section. “Apoptosis (folds)” is the fold increase in apoptosis relative to untreated control.

Cell lines	Treatment	Apoptosis (folds)	Effect of Scavengers
PC3	Untreated	-	
	EGCG 25 µM	2.06	-
	TU	1.32	35.92233
	Catalase	1.89	8.252427
	SOD	1.76	14.56311
MDA-MB-231	Untreated	-	
	EGCG 25 µM	3.1	-
	TU	2.01	35.16129
	Catalase	2.56	17.41935
	SOD	2.64	14.83871
BxPC3	Untreated	-	
	EGCG 25 µM	2.37	-
	TU	1.76	25.7384
	Catalase	2.11	10.97046
	SOD	1.95	17.72152

## Data Availability

Not applicable.
